# *Hypothesis* Holiday sudden cardiac death: food and alcohol inhibition of SULT1A enzymes as a precipitant

**DOI:** 10.1002/jat.2764

**Published:** 2012-06-08

**Authors:** Ken Eagle

**Affiliations:** Katy, TX 77494, USA

**Keywords:** sudden cardiac death, SULT1A inhibition, catecholamines, arrhythmia, phenols, polyphenols

## Abstract

Sudden cardiac death is a significant health issue, causing millions of deaths worldwide annually. Studies have found that the likelihood of such death is higher in winter. Further studies identified that the highest likelihood occurs on Christmas Day and New Years Day, but not the interim period. Thanksgiving, Independence Day and the Islamic holiday Eid Al-Fitr also show significant increases in the rate of cardiac events or death. A number of mechanisms have been proposed, but none have satisfactorily explained the evidence. This article reviews the data supporting the existence of a holiday cardiac death phenomenon, the involvement of catecholamines and the normal modes of human catecholamine deactivation. Further evidence is reviewed that supports a hypothesized mechanism whereby critical SULT1A catecholamine deactivation enzymes can in some patients be inhibited by naturally-occurring phenols and polyphenols in foods and alcohols. If deactivation is inhibited by holiday consumption excesses, holiday stress or excitement could lead to a buildup of catecholamines that can cause fatal arrhythmias. Awareness of this mechanism could reduce deaths, both through doctor/patient education leading to a moderation in consumption and through the potential identification of patients with a predisposition to SULT1A inhibition. This hypothesis also raises parallels between sudden cardiac death in adults and Sudden Infant Death Syndrome (SIDS). The possible involvement of SULT1A inhibition in SIDS is discussed. Copyright © 2012 John Wiley & Sons, Ltd.

## INTRODUCTION

Sudden cardiac death (SCD) is thought to kill over 300 000 people in the US each year (Rubart and Zipes, 2005). A sequence of ventricular tachycardia leading to ventricular fibrillation and then asystole is typically the immediate cause of death, and often occurs in people with no obvious prior signs of heart trouble. A review of SCD discussing potential mechanisms and risk factors (age, smoking etc.) is available (Zipes and Wellens, 1998).

A body of literature, introduced in the following section, has discussed the variation in the frequency of SCD over time. Work identifying a winter peak in SCD led researchers to suspect direct or indirect effects of the cold, but other work finding the same seasonality in Los Angeles and Hawaii undercut this theory. Detailed analysis identified the particular days of Christmas and New Years as having the highest likelihood of SCD. Stress has received scrutiny as a possible cause, given the ability of stress-related catecholamine hormones to trigger SCD, but without a convincing explanation of the relation to SCD seasonality.

An entirely separate set of literature discusses the human SULT1A enzymes (details below). This system plays a key role in deactivating catecholamines, the hormones the body uses to react to stress. These studies document that many plants naturally contain phenols and polyphenols that inhibit SULT1A *in vitro*. There is also evidence that this inhibition occurs *in vivo*, although it is generally not recognized as such.

This hypothesis paper ties together these two existing strands of work. It is proposed that holiday overindulgence in plant-based foods (herbs and spices, recreational alcohols, fruits and vegetables) can act toxicologically to inhibit the SULT1A catecholamine deactivation system in some patients. With reduced deactivation, catecholamines resulting from stress or excitement can accumulate to levels that can trigger arrhythmias and SCD. While it is believed that this diet-based mechanism can cause SCD, it is not claimed to underlie all cases of SCD.

Understanding this potential mechanism would provide a clear benefit to doctors and potential patients, while the level of SULT1A activity could be testable allowing identification of people predisposed to SULT1A inhibition and SCD.

### Sudden Cardiac Death: Background

The likelihood of SCD varies significantly over the course of a year. In Minnesota, the likelihood of SCD was found to be higher in winter (Gerber *et al.*, 2006). Seasonal variation, also peaking in winter, was seen in Hawaii by Seto *et al.* (1998), in spite of a sub-tropical location with little seasonal temperature variability. In Hong Kong, crude death rates from coronary disease were 37% higher at the January peak than during the September trough (Wong *et al.*, 1999). Anand *et al.* (2007) used US data from implanted cardioverter-defibrillators and again saw a winter peak, but also a higher proportion of episodes occurred on Fridays. There was a bimodal circadian distribution peaking from 08.00 to 13.00 hours with a secondary peak from 17.00 to 22.00 hours. Using Australian monthly ischemic heart disease data, Enquselassie *et al.* (1993) also found winter (June to August) peaks in mortality. Events such as cold temperatures (Gerber *et al.*, 2006) and home-team losses in Super Bowls (Kloner *et al.*, 2009) have also been found to increase cardiac deaths.

Data showing generic winter peaks led to more-detailed research, focusing on the holiday season around Christmas and New Years. Death certificate data in Los Angeles county confirmed that coronary artery disease peaked in December/January, but also indicated that deaths around the Christmas and New Year season were even higher than other times during the winter (Kloner *et al.*, 1999). This was later amplified in a day-by-day analysis (Phillips *et al.*, 2004) of all US deaths from mid-1973 to mid-2001. That study found that daily US cardiac sudden deaths peaked on the specific days of Christmas and New Years, with a pronounced dip between the two. A study of deaths in Newcastle, UK, by Milne (2005) found a peak on New Years but not Christmas or Easter. Phillips *et al.* (2010) looked at all US deaths, this time from 1979 to 2004, reconfirming Christmas and New Years Day as the two largest mortality spikes on the basis of dead-on-arrival or emergency department deaths. These days were closely followed by Thanksgiving and Independence Day, whereas the less-celebratory holidays of Labor Day and Memorial Day had lower mortality spikes. Presidents Day did not show a mortality spike on the same emergency basis. Zubaid *et al.* (2006) showed that 6-year admissions to the coronary care unit of a large hospital in Kuwait peaked on the second of the 4 days of Eid Al-Fitr, an Islamic religious holiday marking the end of Ramadan. The holiday is festive, marked by feasts and gifts with friends and family.

A number of potential precipitants are discussed in the articles referenced above, including direct effects of the cold weather, the shoveling of snow, winter darkness, patients going home from hospitals for the holidays but then dying at home, psychological stress of holidays, overcrowded emergency departments, increased travel, postponement of death to reach symbolic occasions, miscoding of death dates, influenza/pneumonia and delays in seeking medical care. None of these explanations have been found to satisfactorily explain the death peaks at celebratory holidays. Another mentioned possibility, changes in diet and alcohol consumption on the holidays, is discussed below.

Stress, whether because of temperature, shoveling snow, increased darkness, holiday travel or family reunions, is a recurrent feature in many of the proposed mechanisms. With stress come the stress hormones dopamine, epinephrine and norepinephrine (the catecholamines). It is not surprising that mechanisms have attempted to link catecholamines to SCD. Catecholamines are known to cause arrhythmias (e.g. Tomaselli and Zipes, 2004), and beta-blockers that inhibit activation of beta-receptors by catecholamines are known to reduce cardiovascular mortality including sudden cardiac death (Gottlieb *et al.*, 1998, Rubart and Zipes, 2005; Tomaselli and Zipes, 2004).

### Catecholamines: Background

It is useful to review how humans produce, use and deactivate catecholamines. Some dopamine can be directly ingested from foods such as bananas. The majority of dopamine is produced by the decarboxylation of L-DOPA, which the body produces by hydroxylation of L-tyrosine (Goldstein, 2001). L-tyrosine is either directly ingested as part of our protein intake, or produced from phenylalanine that is also ingested in protein-rich foods. Dopamine is converted by dopamine beta-hydroxylase to norepinephrine, which in turn can be methylated to epinephrine. The catecholamines act both as neurotransmitters and hormones. In their hormonal roles, they act through the adrenergic receptors to regulate the ‘fight-or-flight’ response, impacting blood pressure, heart rate and breathing rate among other functions (Fitzgerald, 2007).

Three different enzymatic paths deactivate the catecholamines. Two routes, deamination by monoamine oxidase (MAO) and methylation by catechol-*O*-methyltransferase (COMT), were discovered earliest and are perhaps most familiar to practitioners. A recent review by Eisenhofer *et al.* (2004) fully describes these deactivation mechanisms. The third route depends on sulfonation by the cytosolic sulfotransferase (SULT) enzymes SULT1A1 and SULT1A3.

Endobiotics and exobiotics are sulfonated by the SULTs (Strott, 2002), with five families of SULT enzymes existing in mammals. The sulfonate donor in all cases is 3′-phosphoadenosine 5′-phosphosulfate (PAPS). The SULT1 family includes SULT1A (primary substrates phenolics and catecholamines), SULT1B (thyroid hormones), SULT1C (xenobiotics) and SULT1E (estrogenic steroids). Acting as the ‘gut–blood barrier’, the SULT1A enzymes protect humans from ingested catecholamine precursors (Eisenhofer *et al.*, 1999). SULT1A1 preferentially sulfonates planar phenolics, but can also act on dopamine. The role of SULT1A2 is unclear. SULT1A3 and its duplicate SULT1A4 are the key enzymes for sulfonating dopamine and tyrosine. Sulfonation is typically a detoxification pathway as it usually facilitates elimination in the urine by making a molecule more hydrophilic (Hempel *et al.*, 2005).

As mentioned above, most dopamine is formed from L-tyrosine via L-DOPA. Approximately 45% of the dopamine formed in humans is created in the intestines (Eisenhofer *et al.*, 1997). Most is normally immediately deactivated by SULT1A3. The majority of all three catecholamines in humans circulate in the inactive sulfonated form (*c*. 97% of the plasma dopamine, 73% of the plasma norepinephrine and 84% of the plasma epinephrine, are sulfonated) (Strott, 2002).

### Food and Alcohol as a Precipitant of SCD

Diet and alcohol consumption often change on holidays. We eat more. We are more likely to eat food cooked by others. Special ingredients and spices are often included. Many drink alcohol, often much more than during the rest of the year.

Given the spike in holiday deaths, and the changed eating and drinking habits at the holidays, it is natural to hypothesize a link. Kloner (2004) considered weight gain, salt intake and fatty meals, along with excess consumption of alcohol, as possible causes of holiday death. With the exception of alcohol use, it is difficult to reconcile these possibilities with sudden death on particular days. Phillips *et al.* (2004) dismissed the idea that diet and alcohol causes SCD because ‘Inpatients, whose diet and alcohol consumption are strictly regulated, produce a holiday peak’. This statement makes the implicit assumption that hospitals know what foods are unhealthy for cardiac inpatients. Unfortunately, as discussed below, this may be incorrect.

The earlier discussion confirms that stress is present in many forms on holidays. The holidays can also be exciting. The body responds to both stress and excitement with catecholamines, and large holiday meals can certainly provide sufficient L-tyrosine or phenylalanine to source those catecholamines. As discussed above, most of the catecholamines are deactivated in the intestines by sulfonation catalyzed by the SULT1A1 and SULT1A3 enzymes. On a typical celebratory holiday, the body probably produces more catecholamines than on normal days, in response to the extra stress and excitement, and then deactivates those same extra catecholamines.

**Figure 1:**
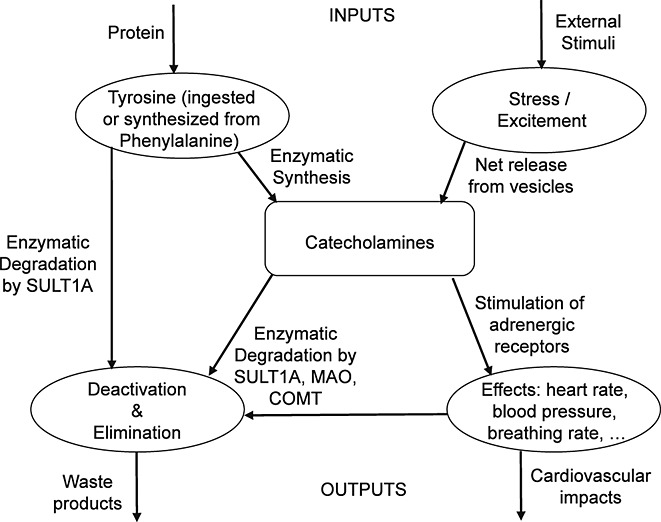
Schematic overview of the catecholamine system. Higher levels of the inputs on holidays lead to higher levels of outputs. Inhibition of SULT1A biases the outputs towards additional cardiovascular impacts.

A high-level overview of this process is shown in Figure 1. The body reacts to inputs of protein and certain external stimuli to create or release catecholamines, which have effects leading to cardiovascular impacts. Enzymatic degradation throughout the process helps to manage the amount and impacts of the catecholamines.

However, consider what would happen if the SULT1A portion of the deactivation mechanism is shut down. Catecholamines would build up from the stress and excitement, and be exposed to less deactivation, leading to additional cardiovascular impacts. It is known that catecholamines in sufficient quantities can trigger the arrhythmias thought to underlie SCD (Dhalla *et al.*, 2010). With inhibited catecholamine deactivation, there is a prescription for trouble.

### SULT1A Inhibition

A number of previous studies (e.g. Bamforth *et al.*, 1993; Coleman, 2010; Coughtrie and Johnston, 2001; Gibb *et al.*, 1987; Littlewood *et al.*, 1985; Nishimuta *et al.*, 2007; Yeh and Yen, 2003) have found evidence of *in vitro* inhibition of SULT1A enzymes by natural phenols and polyphenols in plants. These molecules include quercetin, hesperitin, vanillin, curcumin, resveratrol, quinic acid, caffeic acid, chlorogenic acid and a number of other substances. These various chemicals can be found in most alcohols (especially those from red grapes), most herbs and spices, citrus fruits, berries, coffee, tea, chocolate, onion, tomato and many other common items from plants (Neveu *et al.*, 2010).

Several studies (Boyer *et al.*, 2004; Dulloo *et al.*, 1999; Graham *et al.*, 1998; Heikkonen *et al.*, 1991; Spaak *et al.*, 2008) have demonstrated an increase in plasma free catecholamines after human ingestion of SULT1A inhibitors. Each of these studies controlled for confounding effects of other active substances such as caffeine and ethanol. This is direct evidence of *in vivo* SULT1A inhibition leading to increases in catecholamines. Further evidence comes from a common diagnostic test for excess catecholamines that looks for metanephrine metabolites in plasma or urine. The standard patient warning prior to such testing suggests avoidance of coffee, tea, chocolate, vanilla, bananas and citrus fruits [http://labtestsonline.org/understanding/analytes/urine-metanephrine/tab/sample]. These are all SULT1A inhibitors, suggesting that *in vivo* SULT1A inhibition leads to increased catecholamines that interfere with the test results. Further indirect evidence is provided by studies showing ingestion of SULT1A inhibitors leading to changes in blood pressure (Corti *et al.*, 2002; Edwards *et al.*, 2007; Erlund *et al.*, 2008; Morand *et al.*, 2010) via any of the catecholamines (Fitzgerald, 2007) and the triggering of migraine headaches (Fukui *et al.*, 2008; Littlewood *et al.*, 1988; Peatfield *et al.*, 1984, 1995) via dopamine (Charbit *et al.*, 2010). Nicolodi and Sicuteri (1999) showed that consumption of alcohol was more likely to provoke a migraine during periods of stress, reinforcing the link between inhibitor ingestion (in the alcohol), stress and abnormally-high catecholamines (causing the migraine).

There are non-synonymous single-nucleotide polymorphisms that could make some patients more susceptible to SULT1A inhibition. SULT1A1 has three alleles, with the *2 and *3 alleles having lower sulfonation capability than the normal *1; allele frequency varies by race, with Nagar *et al.* (2006) finding that the higher-activity 1A1*1 occurs with a frequency of 66% in Whites, 48% in African-Americans and 91% in Chinese. African-Americans also have a lower-activity SULT1A3 allele, with a frequency of around 4% (Thomae *et al.*, 2003). Additionally, Marazziti *et al.* (1998) show that SULT1A activity varies seasonally, with significantly higher activities in summer; SULT1A3 is known to be induced by glucocorticoids (Bian *et al.*, 2007), and some studies (e.g. Vondrašová *et al.*, 1997) show higher cortisol levels in summer due to the longer photoperiod.

A diet consistently low in SULT1A inhibitors could also make some patients more susceptible. Although apparently paradoxical, this could cause the SULT1A enzymes to down-regulate, leaving the body exposed when a high dose of SULT1A inhibitors is ingested during a holiday celebration. This could explain why consumption of nuts (high in SULT1A inhibitors) was found by Albert *et al.* (2002) to be inversely correlated with the risk of sudden cardiac death, although the findings were explained in terms of fatty acid content.

A fuller explanation of the *in vivo* inhibition studies, relevant genetic variation and the broader SULT1A-inhibition hypothesis including proposed tests and new approaches is available (Eagle, 2012). That article also hypothesizes that the well-known ‘holiday heart’ syndrome, where non-fatal arrhythmias arise after consumption of large amounts of alcohol, is probably another demonstration of *in vivo* SULT1A inhibition leading to excess catecholamines.

### How was this not Identified Previously?

At first reading, it seems unlikely that common foods are harming people, especially to the extent of sudden death. Over thousands of years mankind has identified numerous toxic plant-based substances. How could a whole class of threatening compounds, existing in many varieties of fruits and vegetables, not have been identified? There are a number of reasons that could explain this.

It is likely that only excessive amounts of food and drink associated with holidays or celebrations are sufficient to trigger these acute effects. The low-activity SULT1A alleles that may predispose some people to this phenomenon are in the minority. Any impacts from recreational alcohols are assumed to be caused by ethanol. The complexity of a two-step mechanism, where exposure to a catecholamine source could occur hours after the initial enzyme inhibition, thereby delaying symptoms significantly from when inhibitor was ingested, makes attribution difficult. Epidemiological studies are not equipped to identify a class of chemicals dispersed throughout a large portion of the food supply.

There are two difficulties with identifying SULT1A inhibition via animal testing. First, most common foods have been eaten for centuries, and are ‘generally recognized as safe’. More importantly, most testing is done in non-primate mammals that have no SULT1A3 ortholog (Riches *et al.*, 2009). Glucuronidation, catalyzed by different enzymes, is the primary route for catecholamine deactivation in such animals (Strott, 2002). Without SULT1A3 to be inhibited, they will not demonstrate these effects. Dietary tests in primates are uncommon.

It is also difficult to trace a mechanism that works by raising catecholamine levels. The normal body is constantly adjusting catecholamine concentrations in response to a variety of stimuli. Any additional effects owing to SULT1A inhibition must somehow be separated from the normal underlying variability.

### Sudden Death of Infants

SCD naturally brings to mind the case of older adults. However, infants also die suddenly, and the subject warrants a brief discussion.

Sudden Infant Death Syndrome (SIDS) is a leading cause of death in children under 1 year of age, and the parallels with the above discussion are striking: the root cause is unknown, arrhythmias may be involved (e.g., Arnestad *et al.*, 2007), a wide variety of stressors (including sleeping position, infection and exposure to nicotine) are hypothesized as risks (Goldwater, 2011) and there is a seasonal pattern to SIDS deaths in Hawaii peaking in winter (Mage, 2004). In light of the SULT1A allele frequencies cited above, it is interesting to note that for US infants above 2500-g birthweight, the 2007 mortality rates due to SIDS were 86.5, 43.6 and 20.1 per 100 000 live births for Black, White, and Asian or Pacific Islander mothers, respectively [http://ftp://ftp.cdc.gov/pub/Health_Statistics/NCHS/Dataset_Documentation/DVS/periodlinked/LinkPE07Guide.pdf]. There are undoubtedly many confounding factors contributing to these differences, but, based on the ethnic distribution of non-*1 alleles cited above, the results are consistent with susceptibility to SULT1A inhibition. Phenolics that could act as SULT1A inhibitors have been found in both breast milk and infant formulas (Li *et al.*, 2009). At least one study (Nowell *et al.*, 2000) has found that males have significantly lower SULT1A1 activity than females, which if confirmed in infants could explain why SIDS strikes males more often than females.

Further research into the possible involvement of SULT1A inhibition in SIDS may be warranted.

## CONCLUSIONS

Sudden cardiac death on holidays is a real phenomenon for which previously-proposed mechanisms have not provided a satisfactory explanation. A mechanism combining a catecholamine source such as stress with diet-based inhibition of the key SULT1A enzymes, possibly aided by genetic predisposition to SULT1A inhibition, appears more likely. This in turn suggests a potential mechanism applicable to SCD in general, based on catecholamines plus reduced SULT1A activity whether due to inhibition or other causes; this could account for the known SCD variability with time of year (higher SULT1A activity in summer), time of day (raised catecholamine levels in morning, inhibition by heavy meal and alcohol in early evening), race (SULT1A allele distribution), gender (lower SULT1A activity and higher catecholamine response in males), and Mondays and other stressful events (catecholamine increases).

If lower-activity SULT1A proves to be associated with SCD, a marker will have been identified that could allow preventive actions or at least targeted education. This should be testable.

The food supply is not unsafe, and overindulgence in plant-based foods and alcohols will not necessarily lead to SCD. However, similar to food intolerances, there are some people who may need to moderate consumption of some foods and alcohols, especially in combination with stress or excitement. Unfortunately, exceeding these limits in this case can lead to sudden death rather than just discomfort. Ongoing work to use phenols and polyphenols as medicinals/antioxidants (e.g. Edwards *et al.*, 2007) should be assessed in light of the potential for acute reactions.

The possible involvement of SULT1A inhibition in SIDS should be studied.

## CONFLICTS OF INTEREST AND SOURCE FUNDING

There are no conflicts of interest. This work was entirely self-funded by the author.
